# The Underexplored Genus *Microbispora*: A Treasure Trove of Secondary Metabolites with Diverse Chemistry, Potent Bioactivities, and Biosynthetic Insights

**DOI:** 10.3390/md24080263

**Published:** 2026-07-29

**Authors:** Mingqi Chen, Qingyun Song, Zhi Zhang, Shaowei Liu, Wongsakorn Phongsopitanun, Chenghang Sun, Hongwei Guo, Qinpei Lu

**Affiliations:** 1 Guangxi Key Laboratory for Bioactive Molecules Research and Evaluation, Guangxi Key Laboratory of Composition-Activity Relationship of Traditional Chinese Medicine, School of Pharmacy, Guangxi Medical University, Nanning 530021, China; 13724066684@163.com (M.C.); qingyun03@outlook.com (Q.S.); zhangz002248@163.com (Z.Z.); 2 Department of Microbial Chemistry, Institute of Medicinal Biotechnology, Chinese Academy of Medical Sciences & Peking Union Medical College, Beijing 100050, China; liushaowei3535@163.com (S.L.); chenghangsun@hotmail.com (C.S.); 3 Department of Biochemistry and Microbiology, Faculty of Pharmaceutical Sciences, Chulalongkorn University, Bangkok 10330, Thailand; wongsakorn.p@chula.ac.th; 4 Key Laboratory of Longevity and Aging-Related Diseases of Chinese Ministry of Education & Center for Translational Medicine, Guangxi Medical University, Nanning 530021, China

**Keywords:** *Microbispora*, secondary metabolites, chemical diversity, biological activities, biosynthetic gene clusters

## Abstract

Rare actinomycetes have emerged as important yet underexplored reservoirs for the discovery of novel bioactive compounds. *Microbispora*, a genus of rare actinomycetes, is widely distributed across diverse ecological niches, including terrestrial soils, marine-associated environments, plant-associated ecosystems, and insect-derived environments. To date, 81 secondary metabolites have been reported from this genus, encompassing quinones, chromones and chromanones, macrolides, other polyketides, alkaloids, peptides and diketopiperazines, and miscellaneous structural classes. These metabolites display antimicrobial, anticancer, neuroprotective, antiviral, plant growth-promoting, and enzyme inhibitory activities. Beyond systematically cataloging these compounds, this review provides an integrated analysis of their structure–activity relationships (SAR), biosynthetic origins, and biological significance. In addition, the biosynthetic potential of *Microbispora* is discussed based on reported genomic studies, highlighting the presence of numerous predicted and poorly characterized biosynthetic gene clusters. This review provides an integrative perspective on *Microbispora* as an underexplored but promising source of structurally diverse and bioactive natural products for drug discovery.

## 1. Introduction

Natural products have long played a pivotal role in drug discovery and continue to provide important leads for the development of new therapeutic agents [1,2]. Among microbial producers, actinomycetes have been particularly productive, yielding a broad range of structurally diverse and biologically active secondary metabolites. These microorganisms represent a major reservoir of bioactive natural products and have contributed substantially to the development of antibiotics for human medicine [3,4]. The genus *Streptomyces*, in particular, has been one of the most prolific sources of antibiotics, providing important drugs such as streptomycin, neomycin, and chloramphenicol [5,6]. However, decades of intensive screening have increased the frequency of rediscovering known metabolites from well-studied *Streptomyces* strains, underscoring the need to explore underinvestigated rare actinomycetes as alternative sources of new bioactive metabolites [7].

In recent years, non-*Streptomyces* actinomycetes, commonly referred to as rare actinomycetes, have attracted growing interest as promising sources of new bioactive metabolites; this designation mainly reflects their less frequent isolation and limited investigation under conventional laboratory conditions rather than their actual abundance in nature [8]. Representative genera such as *Micromonospora*, *Amycolatopsis*, *Microbispora*, *Actinomadura*, *Pseudonocardia*, *Saccharopolyspora*, *Actinoplanes*, *Salinispora*, and *Nonomuraea* have yielded chemically diverse and biologically active secondary metabolites [7,9]. Recent genome mining studies further suggest that rare actinomycetes harbor substantial and still undercharacterized biosynthetic gene cluster (BGC) diversity, supporting their value as alternative producers in natural product discovery [10]. The clinical use of rare-actinomycete-derived antibiotics, including vancomycin, rifamycin-derived drugs, gentamicin, teicoplanin, erythromycin, and fidaxomicin, further highlights their therapeutic significance [7].

The genus *Microbispora*, belonging to the family *Streptosporangiaceae*, was originally described by *Nonomura* and *Ohara* in 1957. Members of this genus are Gram-positive, aerobic, filamentous actinobacteria characterized by the formation of longitudinally paired spores on aerial mycelia [11,12,13]. According to the List of Prokaryotic names with Standing in Nomenclature (LPSN), the genus currently contains 32 listed child taxa, of which 12 are validly published and have correct names, whereas the remainder include synonyms or names without valid standing in nomenclature (https://lpsn.dsmz.de/genus/microbispora, accessed on 28 July 2026). *Microbispora* strains have been isolated from diverse habitats, including terrestrial soils [14,15,16,17], marine-associated environments [18,19,20,21,22,23], plant-associated ecosystems [24,25,26], and insect-associated environments [27]. In particular, marine-associated habitats, including marine sponges, mangrove ecosystems, and other coastal niches, represent promising but underexplored sources of *Microbispora* diversity. A representative example is sponge-associated *Microbispora* sp. OK76, which has yielded structurally unique metabolites, including okichromanone and 1-hydroxyphenazine [22], highlighting the potential of marine-associated isolates for discovering novel bioactive natural products. Since the first reports of phenazine and phenoxazinone metabolites from *Waksmania aerata* (later reclassified as *Microbispora aerata*) in 1964 [28], 81 secondary metabolites have been identified from *Microbispora* strains through our literature survey. These metabolites cover several structural classes, including quinones, chromones and chromanones, macrolides, other polyketides, alkaloids, peptides and diketopiperazines, and miscellaneous small molecules. This review summarizes the chemical diversity and biological activities of *Microbispora*-derived metabolites and further discusses their structure–activity relationships, biosynthetic origins, and potential applications in drug discovery. Collectively, these features highlight *Microbispora* as a promising but still underexplored genus for natural product discovery in the post-genomic era.

## 2. The Secondary Metabolites

### 2.1. Quinones

**Figure 1:**
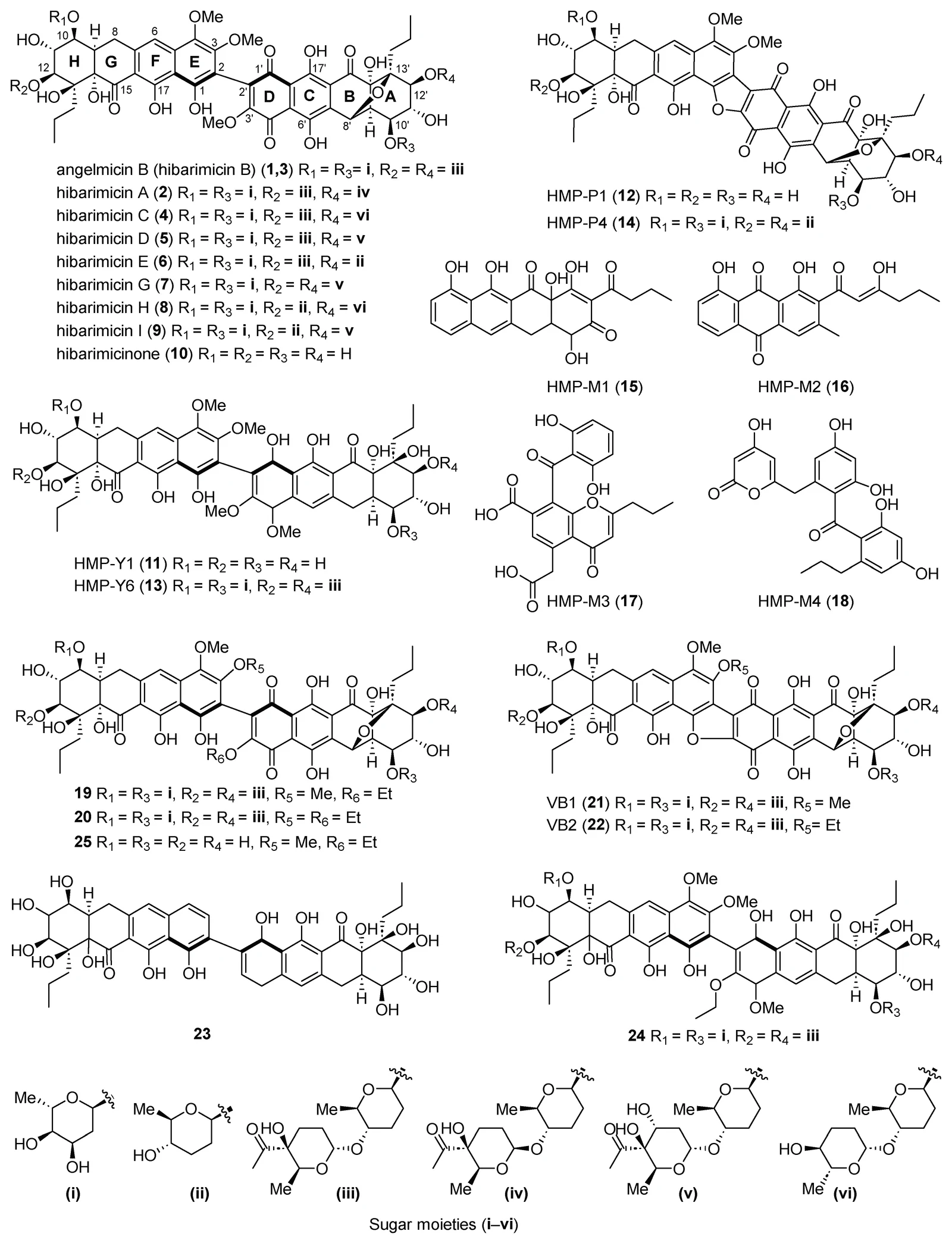
Chemical structures of quinone compounds **1**–**25**. The letters A–H indicate the labels of individual rings within the structures.

Quinones are widely distributed natural products that have been reported to exhibit diverse biological activities, including antimicrobial, anticancer, antioxidant, and anti-inflammatory effects [29,30]. In 1993, angelmicins A and B were isolated from the culture broth of *Microbispora* sp. AA9966, a rare actinomycete obtained from a soil sample collected on Mt. Tennyo, Gunma Prefecture, Japan [16]. Among them, angelmicin B (**1**), a novel inhibitor of oncogenic *src* signal transduction, was subsequently structurally elucidated and is shown in Figure 1. In contrast, the structure of angelmicin A was not fully established in the original reports [16]. The structure of angelmicin B was established by comprehensive spectroscopic analyses, including fast-atom bombardment mass spectrometry (FAB-MS) and extensive one- and two-dimensional nuclear magnetic resonance (NMR) experiments [31]. Angelmicin B was identified as a highly oxygenated phenolic quinone with the molecular formula C_85_H_112_O_37_ and a highly complex pseudo-dimeric architecture consisting of two related polycyclic units joined through a C2–C2′ biaryl bond. Structurally, it contains a highly oxidized naphthylnaphthoquinone chromophore, eight fused six-membered rings, multiple hydroxyl and methoxy substituents, an intramolecular ether bridge, and six deoxyhexose residues. These sugar residues comprise two *α*-digitoxopyranosyl, two *β*-amicetopyranosyl, and two *α*-4-*C*-acetyl-2,3,6-trideoxyhexopyranosyl units, with the digitoxose residues attached to C10/C10′ and the amicetose-containing disaccharide units linked to C12/C12′, respectively [31]. Because of its multiple stereogenic centers and hindered C2–C2′ biaryl axis, the complete stereochemical assignment of angelmicin B remained challenging during its initial structural elucidation and became an important challenge for subsequent synthetic studies. Angelmicin B inhibited the proliferation of multiple human myeloid leukemia cell lines, including ML-1, HL-60, U937, THP-1, HEL, K562, and KU812, with half-maximal inhibitory concentration (IC_50_) values ranging from 0.1 to 1.8 μg/mL [16,32] (Table 1). Notably, it induced differentiation in HL-60 cells along the myelomonocytic lineage, mainly with granulocytic features and some monocytic characteristics, whereas it did not significantly induce differentiation in the other tested leukemia cell lines [32].

Screening of the soil-derived strain *Microbispora rosea* subsp. *hibaria* TP-A0121, isolated from Hibari, Toyama Prefecture, Japan, led to the discovery of hibarimicins A–D and G (**2**–**5** and **7**), a group of structurally related quinone glycosides [33,34]. Additional congeners subsequently reported from this strain included hibarimicin E (**6**), hibarimicins H and I (**8** and **9**), and the common aglycone hibarimicinone (**10**) [35]. Structural investigations revealed that hibarimicin B (**3**) was identical to angelmicin B (**1**), although the two compounds had been independently isolated from different *Microbispora* strains. Hibarimicins share a common highly oxidized naphthylnaphthoquinone aglycone, known as hibarimicinone, while the structural diversity among individual analogs mainly arises from variations in the composition and arrangement of attached deoxysugars [34,35]. Hibarimicins A–D (**2**–**5**) exhibited variable antibacterial activity against Gram-positive bacteria, with some analogs showing strong activity and others displaying moderate activity, as reflected by minimal inhibitory concentration (MIC) values ranging from 0.8 to 12.5 μg/mL (Table 2). However, they were inactive against Gram-negative bacteria (MIC > 100 μg/mL). In addition, these compounds exhibited cytotoxic activity against B16-F10 melanoma and HCT-116 colon cancer cells (IC_50_: 0.7–3.6 μg/mL). Hibarimicins A–D were reported as the first natural glycosides with selective Src tyrosine kinase inhibitory activity without affecting protein kinase A or protein kinase C [33,35]. Subsequent mechanistic investigations provided further insights into the relationship between Src inhibition and cellular differentiation. Hibarimicin B (**3**) was reported as a potent and selective v-Src kinase inhibitor with HL-60 differentiation-inducing activity, whereas its aglycone hibarimicinone (**10**) exhibited stronger but less selective Src inhibition yet failed to induce differentiation. Furthermore, hibarimicin E (**6**) promoted HL-60 cell differentiation despite lacking v-Src kinase inhibitory activity, suggesting that the differentiation-inducing effects of hibarimicins may involve signaling pathways beyond Src kinase inhibition [35].

The biosynthetic pathway of hibarimicins was elucidated through a combination of ^13^C-labeling experiments and cosynthesis studies using blocked mutants. Feeding experiments demonstrated that the hibarimicin carbon skeleton originates from undecaketide-derived precursors that undergo decarboxylation and skeletal rearrangement to form monomeric units, followed by oxidative coupling of these units to generate the dimeric aglycone framework [36]. HMP-Y1 (**11**) was identified as the key biosynthetic intermediate generated through oxidative coupling of two rearranged undecaketide-derived aromatic units; it is subsequently oxidatively modified to hibarimicinone (**10**), the common aglycone of hibarimicins, and then glycosylated to form the HBM complex [37]. In contrast, HMP-P1 (**12**) is considered a shunt metabolite formed through the spontaneous elimination of methanol from hibarimicinone [37,38,39]. Further studies using blocked mutants yielded additional hibarimicin-related metabolites, including HMP-Y6 (**13**), HMP-P4 (**14**), HMP-M1 (**15**), HMP-M2 (**16**), HMP-M3 (**17**), and HMP-M4 (**18**). Although not all of these metabolites are direct pathway intermediates, their characterization provided valuable structural and biosynthetic insights into the hibarimicin pathway and revealed several shunt products arising at different stages of hibarimicin biosynthesis [37,39].

The stereochemical assignment of hibarimicins represented a formidable challenge owing to the coexistence of numerous stereogenic centers and a configurationally stable atropisomeric biaryl axis. In 2004, Hori and co-workers employed a combination of modified Mosher analysis and circular dichroism exciton chirality methods to establish the stereochemistry of the asymmetric centers and assign the atropisomeric C2–C2′ axis as aS in hibarimicins A–K [40]. These studies provided a comprehensive stereochemical framework for the hibarimicin family, which was later corroborated through the total synthesis of key aglycones.

The exceptional structural complexity and potent biological activities of hibarimicins have stimulated extensive synthetic efforts. Early synthetic efforts focused on constructing the AB- and GH-ring systems of hibarimicinone, employing endo-selective Diels–Alder cycloaddition and lipase-mediated kinetic resolution strategies [41]. A major breakthrough was achieved in 2012 when Tatsuta and co-workers reported the first total synthesis of hibarimicinone through a double Michael–Dieckmann cyclization (Hauser annulation) using a thiolactone pseudo-dimer, providing conclusive confirmation of the proposed structure and stereochemistry [42,43]. Independently and shortly thereafter, Liau and co-workers completed the total synthesis of the hibarimicin aglycones, including HMP-Y1, atrop-HMP-Y1, hibarimicinone, atrop-hibarimicinone, and HMP-P1, using a bidirectional annulation strategy featuring a benzyl fluoride Michael–Claisen reaction sequence [44]. This work revealed a pH-dependent rotational barrier around the C2–C2′ biaryl axis and developed biomimetic oxidation and etherification strategies for constructing the highly oxidized frameworks of HMP-Y1, hibarimicinone, and HMP-P1. In addition, a gram-scale enantiospecific synthesis of the A′B′-subunit of angelmicin B was reported, employing a Lewis acid-catalyzed contrasteric Diels–Alder reaction and a tandem silyl zincate 1,6-addition/enolate oxidation sequence [45]. Collectively, these syntheses definitively validated the structural assignments of hibarimicinone and provided strong support for the stereochemical framework established from spectroscopic studies. Despite these advances, the total synthesis of fully glycosylated hibarimicins remains a significant challenge because of their highly complex deoxysugar architectures.

Recent advances in biosynthetic engineering have further expanded access to hibarimicin derivatives. In 2024, the 61 kb hibarimicin BGC, responsible for hibarimicin B biosynthesis, was heterologously expressed in *Streptomyces coelicolor* M1154 [46]. Although initial expression generated only trace amounts of hibarimicin B and resulted in the accumulation of numerous shunt metabolites, rational pathway engineering substantially improved metabolite production and enabled the discovery of a series of previously unreported hibarimicin-related metabolites. These compounds include the 3′-OEt and 3′,3-di-OEt derivatives of hibarimicin B, named 1b (**19**) and 1c (**20**), their ether-bridged analogs VB1 (**21**) and VB2 (**22**), the hibarimicin-related intermediates 12 (**23**) and 15b (**24**). The corresponding aglycone 2b (**25**) was subsequently obtained from 1b (**19**) by acid-catalyzed deglycosylation. Biological evaluation revealed that compound **1c** exhibited the most potent anti-melanoma activity against most of the tested cell lines, with IC_50_ values of 0.3 μM against A375 cells and 2.4 μM against B16-F10 cells. Notably, the glycosyl moieties contributed substantially to the cell-line-dependent activity profile, as deglycosylation to generate **2b** altered the activity profile. These studies established a robust platform for hibarimicin production and provided new opportunities for biosynthetic engineering and SAR investigations of hibarimicin-derived anticancer candidates.

### 2.2. Chromones and Chromanones

**Figure 2:**
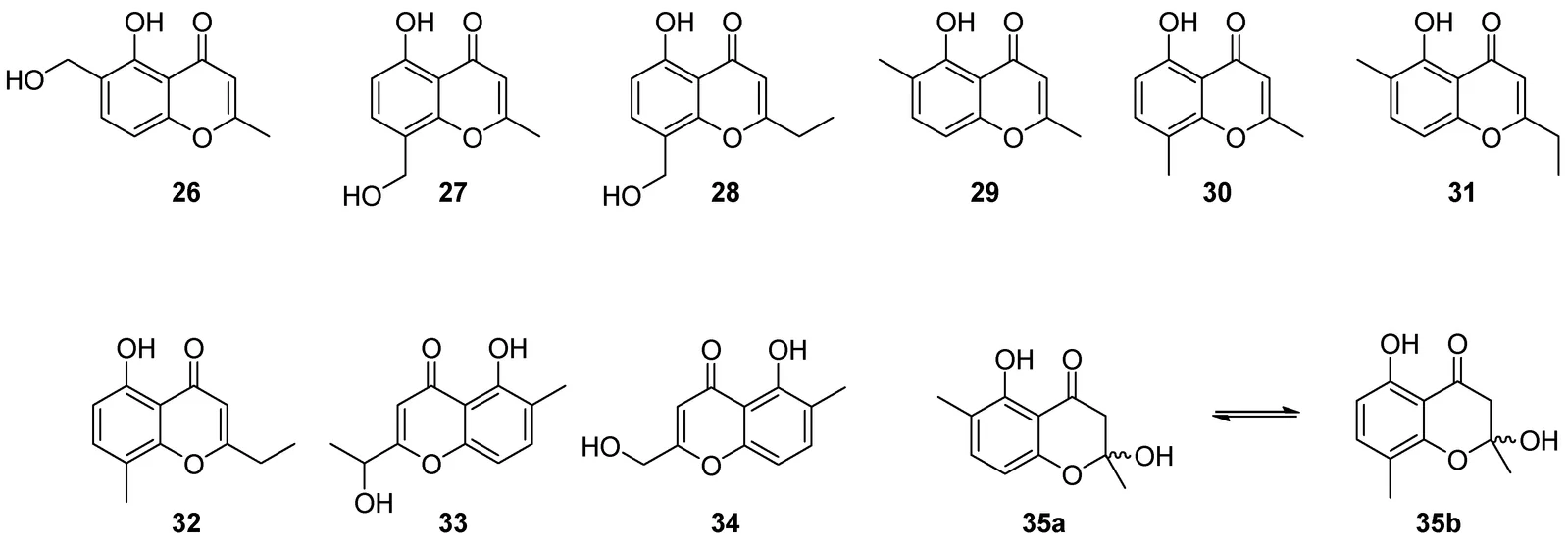
Chemical structures of chromone and chromanone compounds **26**–**35**.

Nine novel chromone analogs (**26**–**34**, corresponding to compounds 1–9 in the original report) were isolated from *Microbispora* sp. TBRC6027, a strain derived from soil collected in an herbal garden in Pathum Thani, Thailand [17], as shown in Figure 2. None of the compounds exhibited antibacterial activities against either Gram-positive or Gram-negative bacteria or cytotoxicity toward human breast cancer MCF-7 cells or Vero cells at the highest tested concentration of 50 μg/mL. Notably, compounds **26**–**27** and **29**–**33** displayed neuroprotective properties under oxidative stress conditions, with P19-derived neuronal cell viabilities ranging from 43.5% to 53.0% at a concentration of 1 ng/mL (Table 3). In addition, compounds **29**, **31** and **32** showed weak cytotoxicity against human small-cell lung cancer NCI-H187 cells, with IC_50_ values ranging from 88.0 to 91.6 μM. A novel chromanone, okichromanone (**35**), was isolated from the culture extract of a marine-derived strain, *Microbispora* sp. OK76, obtained from a sponge collected at Cape Hedo, Okinawa, Japan [22]. Compound **35** was reported as a mixture of two interconvertible isomers, **35a** and **35b**, in an approximately 3:2 ratio. Because these isomers can interconvert through hemiketal ring opening and recyclization, both forms were considered racemic mixtures, and their absolute configurations were not assigned in the original report. Furthermore, okichromanone exhibited antiviral activity against herpes simplex virus type 1 (HSV-1) and herpes simplex virus type 2 (HSV-2), with IC_50_ values of 40 μM and 59 μM, respectively. The *Microbispora*-derived chromone and chromanone metabolites discussed in this section display distinct bioactivity profiles. Most of these compounds showed no detectable antimicrobial activity or nonspecific cytotoxicity under the reported assay conditions, whereas several chromone analogs exhibited neuroprotective activity and okichromanone showed antiviral activity against HSV-1 and HSV-2. These findings highlight their diverse biological properties and suggest that specific chromone substitution patterns may warrant further investigation for neuroprotective potential.

### 2.3. Macrolides

**Figure 3:**
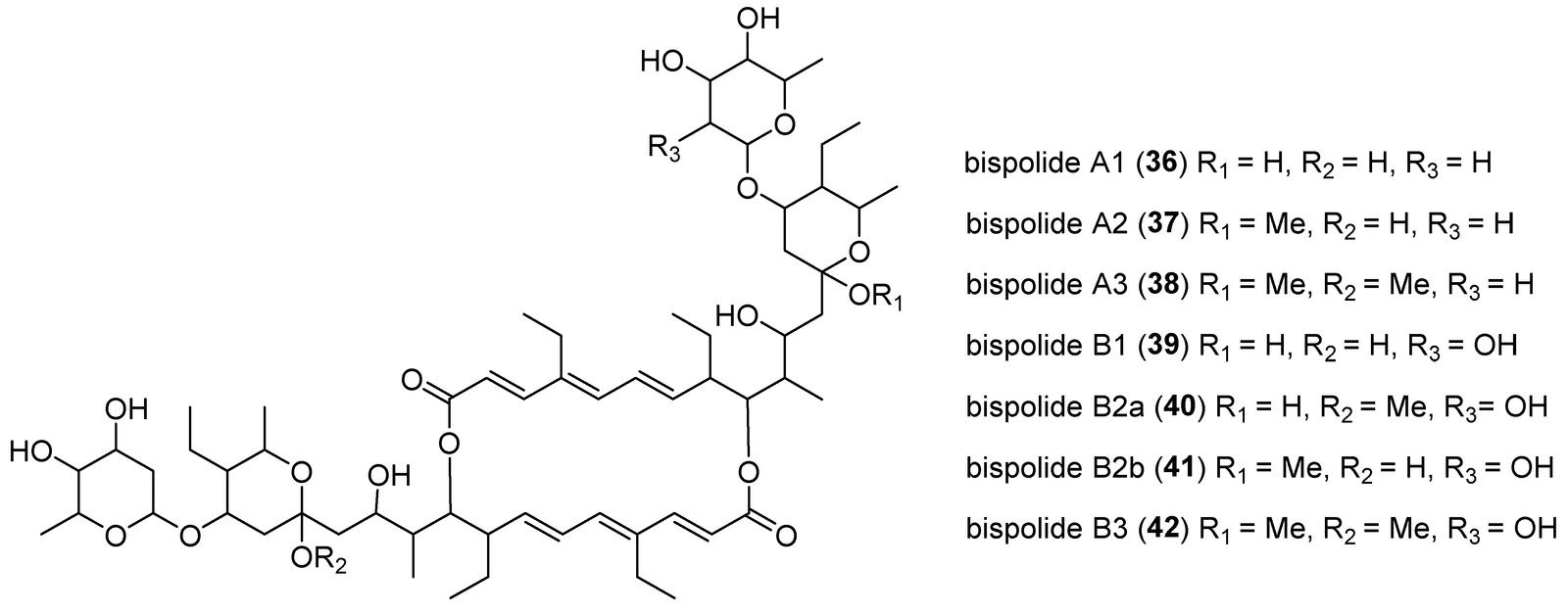
Chemical structures of macrolide compounds **36**–**42**.

Macrolides are an important class of microbial secondary metabolites, exhibiting a variety of biological activities such as antibacterial, anticancer, and antiparasitic effects [47]. In 2007, seven novel 20-membered ring macrolides containing two conjugated trienes and a symmetrical cyclic dimeric structure were discovered in the culture broth of *Microbispora* sp. A34030, a strain isolated from a soil sample collected at a forest station of the Forest Research Institute in Selangor, Malaysia. These compounds were named bispolides A1–A3 (**36**–**38**), B1 (**39**), B2a (**40**), B2b (**41**), and B3 (**42**) [48] (Figure 3). It should be noted that only the planar structures of the original bispolides (**36**–**42**) were established, whereas their complete stereochemical assignments, including the absolute configurations of the sugar moieties, were not reported. Bispolides A1 and B1 exhibited inhibitory effects against Gram-positive bacteria, especially clinically isolated methicillin-resistant *Staphylococcus aureus* (MRSA) strains, with MIC values ranging from 1 to 4 μg/mL. As rare examples of 20-membered macrodiolides from *Microbispora*, bispolides possess unique symmetrical structures and notable anti-MRSA activity, making them promising candidates for further SAR studies.

Subsequent studies revealed that bispolides also possess potent antiprotozoal activities, including antimalarial and antitrypanosomal effects [49]. The naturally occurring bispolides (**36**, **38**, **39**, and **42**) tested in this study showed IC_50_ values of 260–480 ng/mL against the drug-resistant *Plasmodium falciparum* K1 strain, within the same order of magnitude as chloroquine, and IC_50_ values of 57–150 ng/mL against *Trypanosoma brucei brucei*, representing 10–40-fold greater potency than the clinical drugs suramin and eflornithine. The MRC-5/*T. b. brucei* selectivity index reached up to 62. In vivo, bispolide B3, administered intraperitoneally at 25 mg/kg once daily for four consecutive days, slightly extended the mean survival time of *T. b. brucei*-infected mice to 9.5 days compared with 7 days in untreated controls. SAR analysis indicated that the hexose moieties at C-15/C-15′ and the conjugated trienes within the 20-membered ring are important determinants of antiprotozoal activity. These findings highlight the potential of bispolides as lead structures for the further development of antiprotozoal agents.

### 2.4. Other Polyketides

**Figure 4:**
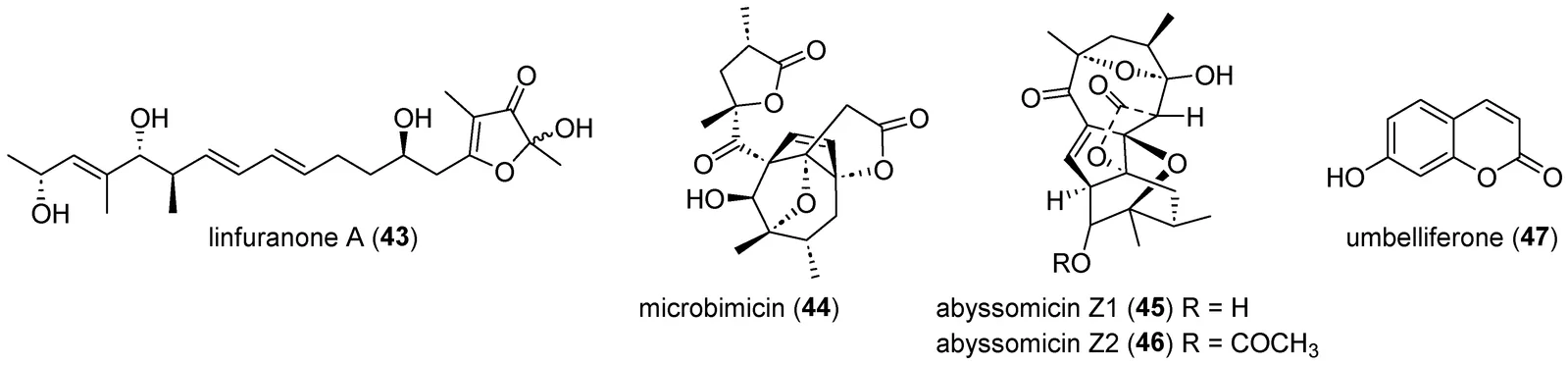
Chemical structures of other polyketide-derived compounds **43**–**47**.

Linfuranone A (**43**), a novel furanone-containing polyketide, was produced by the endophytic strain *Microbispora* sp. GMKU 363, which was isolated from the root tissue of *Lin Ngu Hao*, a Thai medicinal plant collected in Chachoengsao, Thailand [50] (Figure 4). The stereochemical assignment of linfuranone A, including the configuration of its conjugated double bonds, was subsequently supported by the structural elucidation of linfuranone B and its hydrolytic conversion to naturally occurring linfuranone A [51]. Treatment with 50 μM linfuranone A induced differentiation of approximately 47% of mouse ST-13 pre-adipocytes into mature adipocytes, accompanied by lipid droplet accumulation, suggesting its potential relevance to antidiabetic and antiatherogenic effects. Furthermore, compound **43** did not show significant antimicrobial or cytotoxic activities under the reported assay conditions. Notably, a recent biosynthetic study revealed the role of LfnO1, an FAD-dependent monooxygenase involved in furanone formation in linfuranone A and related natural products [52]. LfnO1 catalyzes the molecular skeletal editing of *α*-pyrone substrates into furanone products through a ring-contraction process involving Baeyer–Villiger oxidation, decarboxylation, selective dehydrogenation, and cyclization. This finding reveals an unconventional biosynthetic strategy for furanone formation and highlights the potential application of LfnO1 as a biocatalytic tool for molecular skeletal editing.

*Microbispora rhizosphaerae* TBRC6028, tentatively proposed in 2021 as a novel species of the genus *Microbispora*, was isolated from the rhizosphere soil of *Curcuma longa* in Nakhon Nayok, Thailand [53]. Three new abyssomicin-related compounds, namely microbimicin (**44**), abyssomicin Z1 (**45**), and abyssomicin Z2 (**46**), were isolated from this strain [53]. Their structures were elucidated by extensive NMR spectroscopic analyses, and their absolute configurations were established by single-crystal X-ray diffraction analyses combined with electronic circular dichroism (ECD) spectral data. In particular, the absolute configurations of compounds **44** and **45** were confirmed by X-ray crystallographic analyses, whereas that of compound **46** was assigned based on comparison of its ECD spectrum with that of abyssomicin Z1. A plausible biosynthetic pathway for compounds **44**–**46** was proposed, involving dehydration, reduction, elimination, intramolecular Diels–Alder cycloaddition, epoxidation, and Michael addition reactions. Abyssomicins are a family of macrocyclic spirotetronate polyketides characterized by a tetronate-fused oxabicyclo[2.2.2]octane core and an 11-membered ring system. They have been mainly reported from actinomycetes of the genera *Verrucosispora* and *Streptomyces* [54]. Notably, this study represented the first report of abyssomicin-related compounds from the genus *Microbispora*. Compounds **44** and **45** showed weak antibacterial activity against *Bacillus cereus,* with MIC values of 25 and 50 μg/mL, respectively, and exhibited no cytotoxicity against MCF-7, NCI-H187, or Vero cells at a concentration of 50 μg/mL. In contrast, compound **46** was inactive against *B. cereus* but displayed cytotoxicity against NCI-H187 and Vero cells, with IC_50_ values of 4.6 μM and 38.9 μM, respectively. Compounds **44**–**46** showed no inhibitory activity against *Mycobacterium tuberculosis*, *Acinetobacter baumannii*, *Alternaria brassicicola*, or *Colletotrichum acutatum* at 50 μg/mL.

Umbelliferone (**47**), a coumarin derivative, was obtained from the culture extract of *Microbispora* sp. AL22, a strain recovered from the rhizosphere soil of *Alpinia galanga* in Nakhon Pathom, Thailand. However, compound **47** showed no significant antibacterial or cytotoxic activity against the tested microorganisms and cancer cell lines under the reported assay conditions [55]. Along with the abyssomicin derivatives described above, this finding further expands the known chemical diversity of the genus *Microbispora* and highlights its biosynthetic potential as an underexplored source of secondary metabolites.

### 2.5. Alkaloids

**Figure 5:**
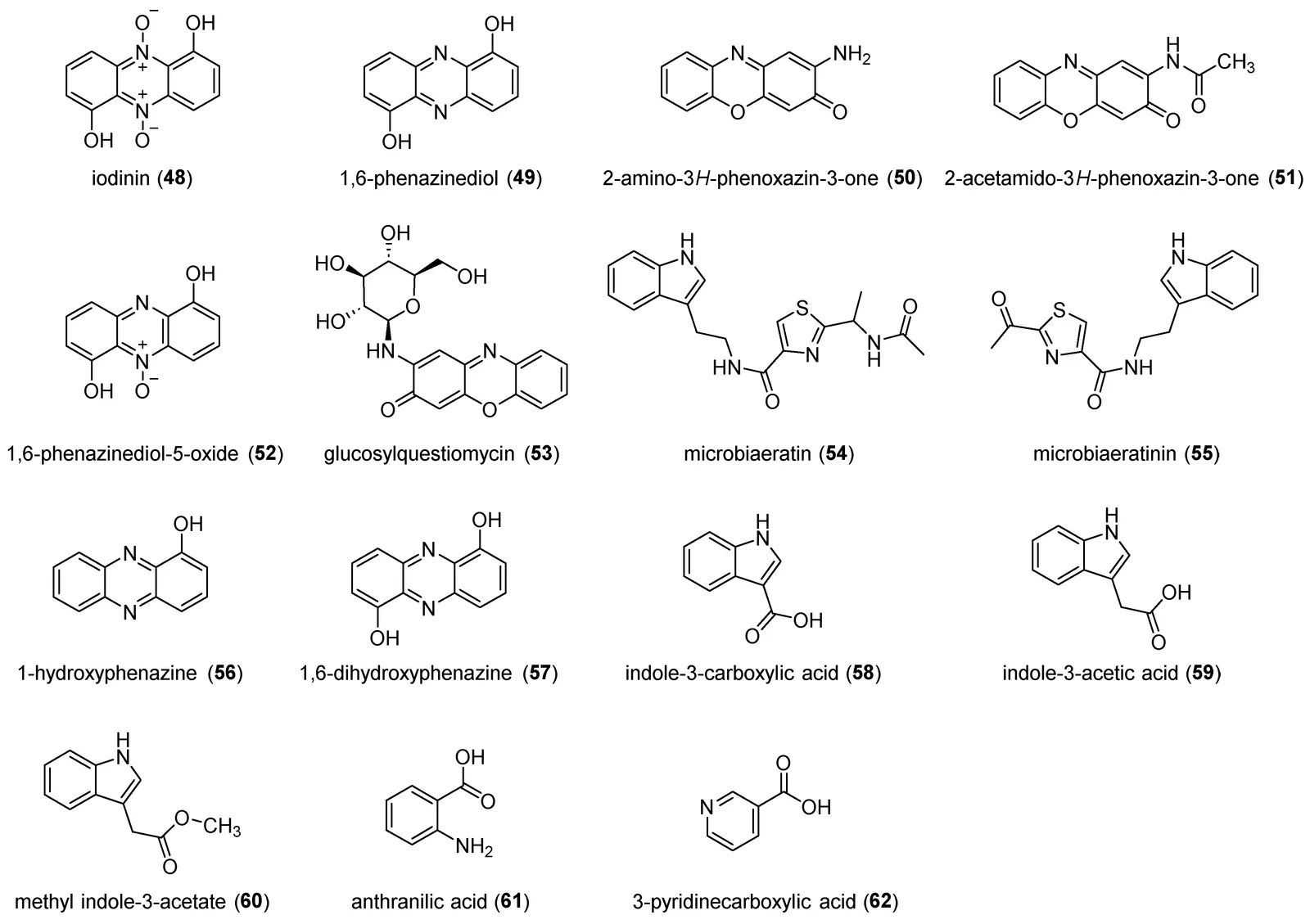
Chemical structures of alkaloid compounds **48**–**62**.

In addition to typical alkaloids, several structurally related nitrogen-containing aromatic metabolites have also been reported from members of the genus *Microbispora*. Although not all of these compounds are classified as alkaloids in a strict sense, they are included in this section because of their structural similarity and the presence of nitrogen-containing aromatic scaffolds. In 1964, four phenazine and phenoxazinone metabolites, iodinin (1,6-phenazinediol-5,10-dioxide, **48**), 1,6-phenazinediol (**49**), 2-amino-3*H*-phenoxazin-3-one (**50**), and 2-acetamido-*3H*-phenoxazin-3-one (**51**), were isolated from *Waksmania aerata* sp. nov., an actinomycete strain obtained from French soil and later reclassified as *Microbispora aerata* due to the taxonomic synonymy between *Waksmania and Microbispora* [28], as shown in Figure 5. This study represented one of the earliest reports of secondary metabolites from the genus *Microbispora*, and phenazines (**48** and **49**) and phenoxazinones (**50** and **51**) represented relatively uncommon classes of microbial metabolites at the time of their discovery. All four compounds exhibited antimicrobial activity against selected Gram-positive bacteria, fungi, and actinomycetes, whereas they were inactive against the tested Gram-negative bacteria. Among them, iodinin (**48**) displayed the strongest activity. One year later, the same research group reported another phenazine derivative, 1,6-phenazinediol-5-oxide (**52**), from *M. aerata* P-132, which was isolated at a yield of approximately 50 μg/L [56]. Compound **52** was proposed to be a biosynthetic intermediate in the conversion of 1,6-phenazinediol (**49**) to iodinin (**48**) and was also suggested to participate in the microbial reduction pathway of iodinin. Its antimicrobial activity was generally intermediate between those of iodinin (**48**) and 1,6-phenazinediol (**49**) [56].

An unusual *N*-glucopyranoside derivative of questiomycin A, glucosylquestiomycin (**53**), together with the known compound questiomycin A (also known as 2-amino-3*H*-phenoxazin-3-one, **50**), was isolated from the fermentation broth of *Microbispora* sp. TP-A0184, a strain obtained from a soil sample collected at Kureha, Toyama, Japan [57]. The sugar moiety of glucosylquestiomycin was determined to possess the D-configuration by chemical synthesis and comparison with synthetic D- and L-isomers. Compound **53** inhibited the growth of selected Gram-positive bacteria, Gram-negative bacteria, and yeasts, with MIC values ranging from 6.25 to 50 μg/mL for susceptible strains, although its potency was lower than that of questiomycin A (**50**). In comparison, questiomycin A (**50**) showed stronger antimicrobial activity against the tested bacteria and yeasts, with MIC values of 1.56–25 μg/mL against bacteria and yeasts. The L-isomer of compound **53**, L-glucosylquestiomycin, lacked activity against Gram-positive bacteria and *Escherichia coli* but retained activity against *Pseudomonas aeruginosa* and yeasts. Moreover, glucosylquestiomycin, L-glucosylquestiomycin**,** and questiomycin A showed cytotoxicity against U937 cells, with IC_50_ values of 11.2, 12.5, and 0.2 μg/mL, respectively. The reduced antibacterial and cytotoxic activities of glucosylquestiomycin (**53**) compared with questiomycin A (**50**) suggest that *N*-glycosylation may attenuate bioactivity, probably by reducing cell-membrane permeability. This finding further underscores the importance of the questiomycin A aglycone pharmacophore for antimicrobial and cytotoxic activity.

In 2007, a new sulfur-containing indole alkaloid, microbiaeratin (**54**), together with the known compound microbiaeratinin (also known as bacillamide, **55**), was isolated from *Microbispora aerata* IMBAS-11A, obtained from penguin excrements collected on Livingston Island, Antarctica [20]. These compounds represent sulfur-containing indole alkaloids featuring a thiazole-carboxamide moiety, an unusual structural motif in natural products. Compound **54** showed weak cytotoxic activity against L-929 mouse fibroblast cells, K-562 human leukemia cells, and HeLa human cervix carcinoma cells. In addition, it showed no activity against common pathogens such as *Bacillus subtilis*, *Staphylococcus aureus*, and *Escherichia coli* at a concentration of 50 μg/disk, and exhibited no inhibitory effect against *Chlorella vulgaris* and *Scenedesmus subspicatus* at 100 μg/mL.

Seven nitrogen-containing metabolites, including 1-hydroxyphenazine (**56**), 1,6-dihydroxyphenazine (**57**), indole-3-carboxylic acid (**58**), indole-3-acetic acid (**59**), methyl indole-3-acetate (**60**), anthranilic acid (**61**), and 3-pyridinecarboxylic acid (**62**), were obtained from the ethyl acetate extract of the fermentation broth of *Microbispora* sp. CSR-4, subsequently identified as *Microbispora hainanensis* [58]. Among these known metabolites, 1-hydroxyphenazine (**56**) has previously been reported to exhibit antifungal activity against *Candida albicans,* with an IC_50_ value of 31.3 μg/mL [59], whereas anthranilic acid (**61**) has been reported to inhibit the spore germination of the phytopathogenic strain *Streptomyces* sp. B-9-1, with an IC_50_ value of 50 μg/mL [60]. Additionally, 1-hydroxyphenazine (**56**) was subsequently isolated, together with okichromanone (**35**), from the culture extract of sponge-derived *Microbispora* sp. OK76. This compound also exhibited antiviral activity against HSV-1 and HSV-2, with IC_50_ values of 86 μM and 123 μM, respectively [22].

### 2.6. Peptides and Diketopiperazines

**Figure 6:**
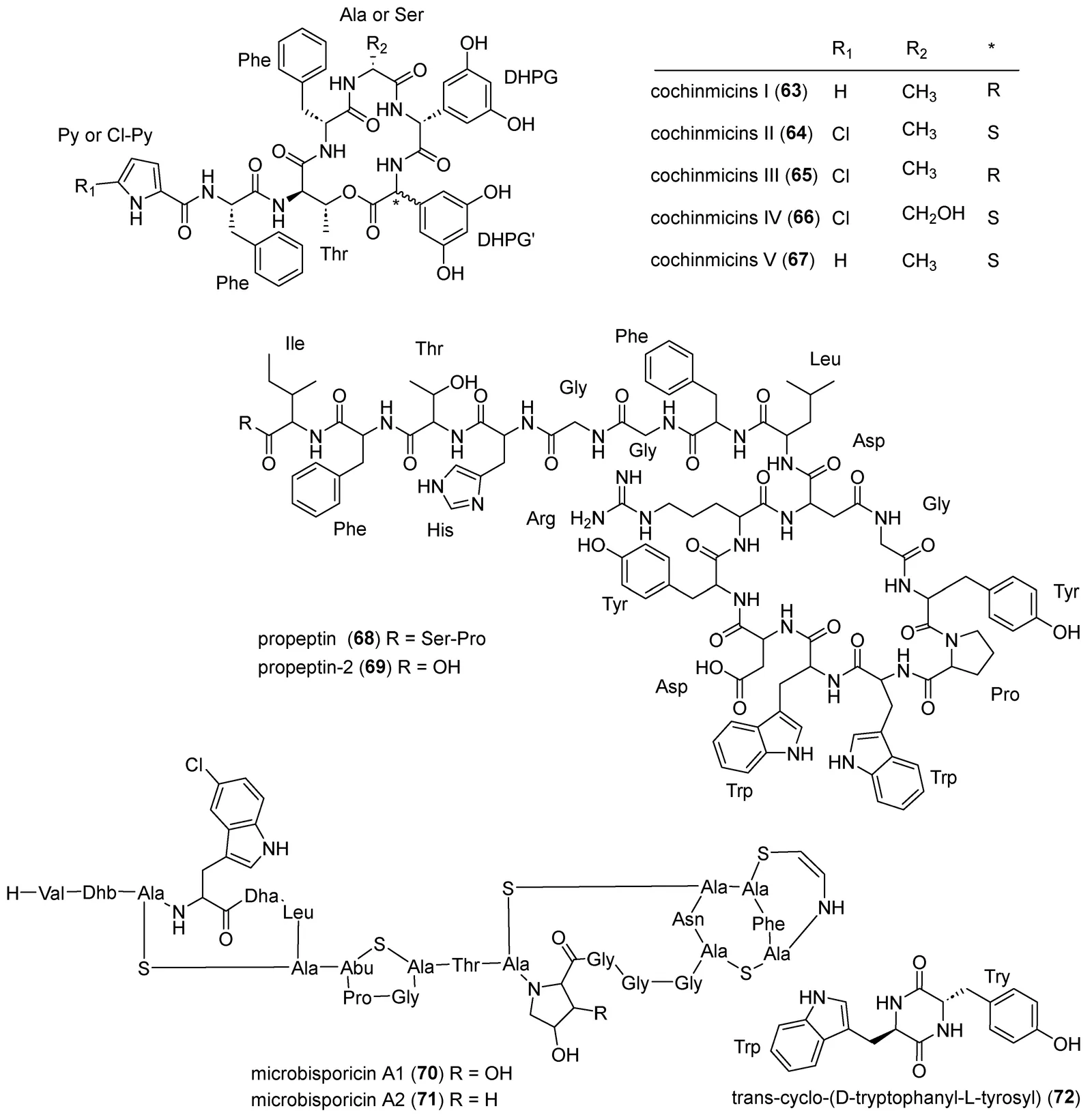
Chemical structures of peptide and diketopiperazine compounds **63**–**72**. The asterisk (*) indicates the stereochemical configuration.

A series of cyclic depsipeptides, cochinmicins I-V (**63**–**67**), were isolated from submerged fermentation cultures of *Microbispora* sp. ATCC 55140, a strain obtained from a soil sample collected in Cochin, India [61,62] (Figure 6). Structural elucidation using MS and MS/MS, 1D and 2D NMR spectroscopy, hydrolysis and methanolysis experiments, and chiral LC analyses revealed that these compounds share a closely related macrocyclic depsipeptide scaffold containing D-*allo*-Thr, L-Phe, D-Phe, two 3,5-dihydroxyphenylglycine (DHPG) residues, and 5-chloropyrrole-2-carboxylic acid (Cl-Py) or pyrrole-2-carboxylic acid (Py). Cochinmicins I–III (**63**–**65**) and V (**67**) contain D-Ala, whereas cochinmicin IV (**66**) contains D-Ser in place of D-Ala. Cochinmicin I is the deschloro analog of cochinmicin III, cochinmicin V is the deschloro analog of cochinmicin II, and cochinmicins II and III differ in the configuration of one DHPG residue [63]. These compounds were identified as competitive endothelin (ET) receptor antagonists. Binding assays showed that cochinmicins I–V inhibited [^125^I]ET-1 binding to ET-A-rich aortic membranes and ET-B-rich rat hippocampal membranes, with IC_50_ values ranging from 0.24 to 90 μM and K_i_ values ranging from 0.12 to 3.1 μM. Among them, cochinmicin I (**63**) showed the highest potency, inhibiting ET-1 binding to rat aortic membranes with an IC_50_ value of 0.24 μM and a K_i_ value of 0.12 μM. In addition, cochinmicin I inhibited ET-1-induced phosphatidylinositol turnover in rat atrial tissue in a dose-dependent manner, with an EC_50_ value of 10.8 μM. Collectively, these compounds inhibited ET-1 binding at both ET-A- and ET-B-associated sites, with cochinmicin I displaying the highest potency as a nonselective endothelin receptor antagonist. In contrast, the cochinmicins exhibited little or no antimicrobial activity; in particular, cochinmicins IV and V were inactive against the tested bacteria, fungus, and mycoplasma at 20 μg/disk [61,62,63].

A novel cyclic peptide antibiotic, propeptin (**68**), was isolated from the mycelium of *Microbispora* sp. SNA-115, a strain obtained from a soil sample collected in Kanagawa, Japan [64,65]. Propeptin consists of 19 amino acid residues, including 16 L-configured amino acids and three glycine residues. It possesses a unique architecture comprising a nine-residue cyclic moiety and a ten-residue linear side chain, with cyclization occurring through an amide bond between the *α*-amino group of Gly^1^ and the *β*-carboxyl group of Asp^9^. Compound **68** was identified as a competitive inhibitor of prolyl endopeptidase (PEP, also known as prolyl oligopeptidase or post-proline-cleaving enzyme), with an IC_50_ value of 1.1 μM and a K_i_ value of 0.7 μM. In addition, it showed no detectable cytotoxicity against the KB and L1210 tumor cell lines at 1000 μg/mL and exhibited weak antimicrobial activity against *Pseudomonas aeruginosa* N-10 L-form, *Mycobacterium phlei* IFO 3158, and *Xanthomonas oryzae* IFO 3312 at 40 μg/disk, producing inhibition zones of 10.6, 14.5, and 11.5 mm, respectively. In 2007, a new propeptin analog, propeptin-2 (**69**), was isolated from the culture broth of the same propeptin-producing strain, *Microbispora* sp. SNA-115 [66]. Compared with propeptin, propeptin-2 lacks the two C-terminal amino acid residues Ser-Pro and therefore consists of 17 amino acid residues. It retained comparable competitive inhibitory activity against prolyl endopeptidase, with a *K*_i_ value of 1.5 μM. In contrast, propeptin-2 showed no detectable antimicrobial activity against *M. phlei* even at 4 mg/disk, representing a more than 100-fold reduction in antimicrobial potency. These findings suggest that the C-terminal Ser–Pro residues are not required for PEP inhibition but may contribute to membrane permeability and/or interactions with molecular targets involved in the antimicrobial effect.

Microbisporicin (NAI-107), a potent lantibiotic peptide, was discovered from the culture broth of the actinomycete *Microbispora* sp. ATCC PTA-5024 as two structurally related components, microbisporicin A1 (**70**) and microbisporicin A2 (**71**), which are typically produced at a ratio of approximately 60:40 [67]. Both peptides consist of 24 amino acid residues containing five intramolecular thioether bridges, including lanthionine-, methyllanthionine-, and AviCys-containing rings, with molecular masses of 2246 Da (**70**) and 2230 Da (**71**), respectively. Remarkably, microbisporicin contains unprecedented post-translational modifications among lantibiotics, including 5-chlorotryptophan and hydroxylated proline residues (4-hydroxyproline in A2 and 3,4-dihydroxyproline in A1). These structural features distinguish microbisporicin from previously characterized lantibiotics. Microbisporicin demonstrated potent antibacterial activity against clinically important Gram-positive pathogens, including several multidrug-resistant bacteria such as MRSA, vancomycin-intermediate-resistant *Staphylococcus aureus* (VISA), and vancomycin-resistant enterococci (VRE), with MIC values ranging from ≤0.13 to 4.0 μg/mL. Mechanistic studies demonstrated that microbisporicin selectively inhibits peptidoglycan biosynthesis, resulting in the accumulation of cytoplasmic UDP-linked peptidoglycan precursors, while exerting limited effects on DNA, RNA, and protein synthesis. In a murine septicemia model caused by *Staphylococcus aureus* Smith 819 ATCC 19636, microbisporicin displayed protective efficacy with a median effective dose (ED_50_) value of 2.1 mg/kg after intravenous or subcutaneous administration, and no mortality was observed at doses of ≥200 mg/kg, suggesting low acute toxicity. Owing to its unique chemical modifications, potent activity against multidrug-resistant Gram-positive pathogens, and favorable preliminary in vivo efficacy, microbisporicin represents one of the most promising lantibiotic candidates discovered from the genus *Microbispora*.

A new natural diketopiperazine (cyclic dipeptide), *trans-*cyclo-(D-tryptophanyl-L-tyrosyl) (**72**), was isolated from the culture broth of *Microbispora aerata* IMBAS-11A, which was obtained from penguin excrements collected on Livingston Island, Antarctica [21]. The structure and absolute configuration of this compound were elucidated by extensive MS and 1D/2D NMR analyses combined with chiral amino acid analysis. This diketopiperazine had previously been reported only as a synthetic product by Sloper and Land (1980) and Zeng et al. (2005) [68,69]. It exhibited low antiproliferative and cytotoxic effects on L-929, K-562, and HeLa cells and showed no inhibitory activity against *Bacillus subtilis*, *Staphylococcus aureus*, *Streptomyces viridochromogenes*, *Escherichia coli*, *Candida albicans*, and *Mucor miehei* at a concentration of 50 μg/disk.

### 2.7. Other Types of Compounds

**Figure 7:**
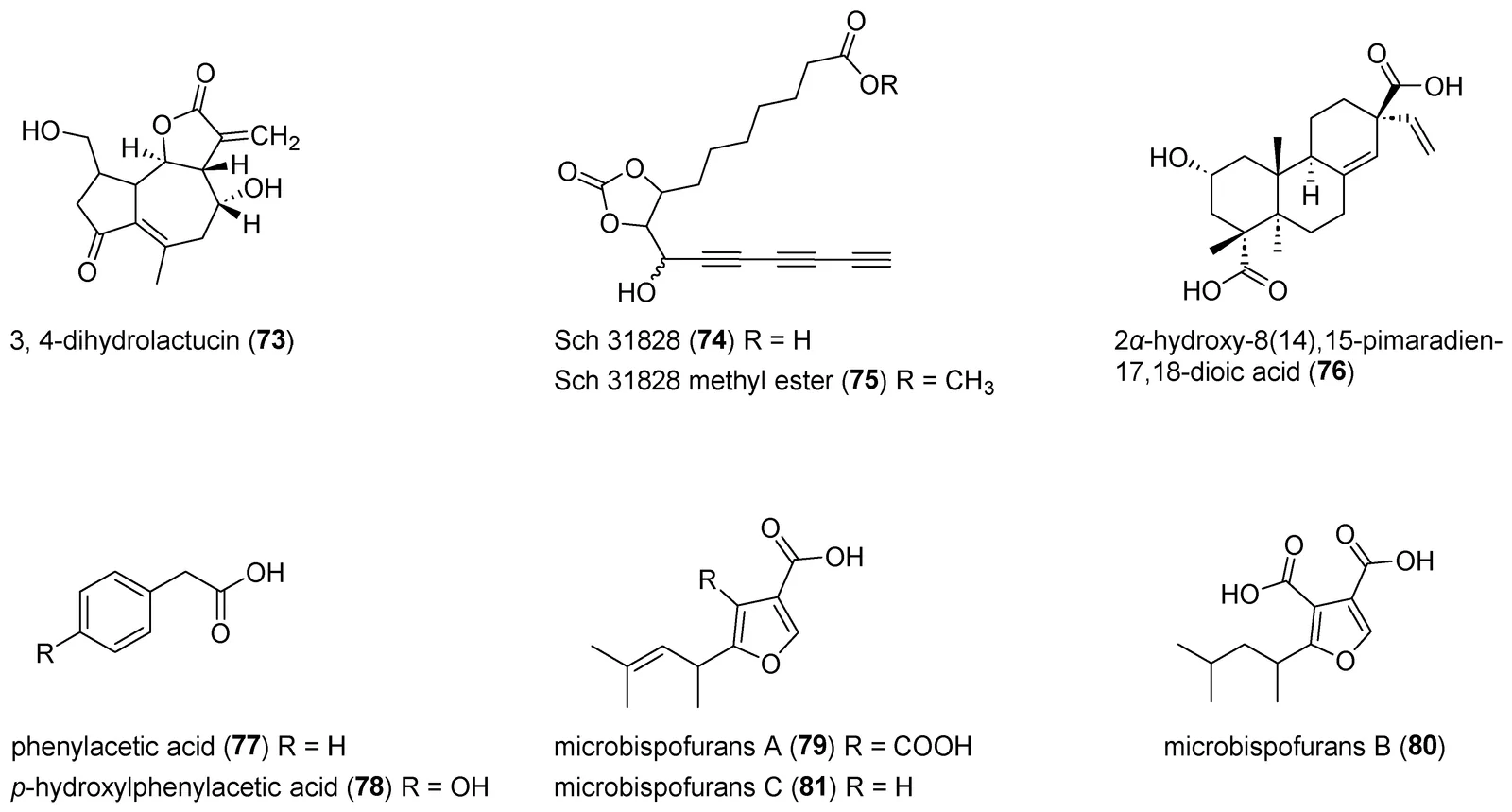
Chemical structures of other types of compounds **73**–**81**.

A sesquiterpene lactone, 3,4-dihydrolactucin (**73**), together with umbelliferone (**47**), was isolated from the endophytic actinomycete *Microbispora* sp. AL22 associated with *Alpinia galanga* [55] (Figure 7). Compound **73** showed antibacterial activity against several pathogenic strains, including *Bacillus cereus*, *B. subtilis*, *Staphylococcus aureus*, MRSA, *Pseudomonas aeruginosa* and *Escherichia coli*, with MIC values ranging from 16 to 64 μg/mL and minimum bactericidal concentration (MBC) values ranging from 64 to 256 μg/mL. Moreover, compound **73** exhibited anticancer activity against MDA-MB-231 and HeLa cell lines, with IC_50_ values of 37.6 and 75.3 μg/mL, respectively. Further mechanistic studies by *Taechowisan* et al. demonstrated that compound **73** induces apoptosis in MDA-MB-231 cells, likely through mitochondrial depolarization and activation of caspase-3 and caspase-8 [70].

A new polyacetylene-containing antibiotic, Sch 31828 (**74**), was isolated from *Microbispora* sp. SCC 1438. Its structure is characterized by a unique dioxolone ring system and a polyacetylene-containing side chain bearing conjugated alkyne units [71]. Due to its instability toward light, pH variation and heat, its methyl ester derivative, Sch 31828 methyl ester (**75**), was prepared. The absolute configuration of Sch 31828 and its methyl ester derivative was not determined in the original report. Compound **75** exhibited potent antifungal activity against *Candida* species and dermatophytic fungi, with geometric mean MIC values of 0.034 and 1 μg/mL, respectively, while displaying moderate activity against *Staphylococcus* strains, with a geometric mean MIC of 29.1 μg/mL. Additionally, three metabolites, including a new diterpene, named 2α-hydroxy-8(14),15-pimaradien-17,18-dioic acid (**76**) and two known phenylacetic acid derivatives, phenylacetic acid (**77**) and *p*-hydroxylphenylacetic acid (**78**), were isolated from the fermentation broth of *Microbispora* sp. CSR-4 [58]. Compound **76** displayed a suppressive effect on recombinant human acetylcholinesterase (rhAChE), with an IC_50_ value of 96.9 ± 2.3 μg/mL, and protected P19-derived neuronal cells against serum deprivation-induced oxidative stress without detectable neurotoxicity.

Recently, three new alkyl/alkenyl furancarboxylic acids, microbispofurans A–C (**79**–**81**), were isolated from plant root-derived *Microbispora* sp. RD004716 [24]. Compounds **79** and **81** exhibited no antimicrobial activity against bacteria and yeasts or cytotoxicity against P388 cells under the tested conditions, compound **80** exhibited only weak antifungal activity against *Saccharomyces cerevisiae* and weak cytotoxicity against P388 cells. Notably, all three compounds promoted plant growth, with treatment of germinated seeds at 10 µM resulting in approximately 62%, 85%, and 102% growth promotion compared to the control, respectively.

**Table 1:** Representative cytotoxic activities of selected *Microbispora*-derived metabolites.

Compound	Class	Producing Strain	Source	Tested Cell Line/System	Representative Activity	Refs.
Angelmicin B (**1**)	Quinones	*Microbispora* sp. AA9966	Soil, Gunma, Japan	ML-1, HL-60, U937, THP-1, HEL, K562, and KU812 cells	IC_50_ 0.1–1.8 μg/mL	[16,32]
Hibarimicins A–D (**2**–**5**)	Quinones	*M. rosea* subsp. *hibaria* TP-A0121	Soil, Toyama, Japan	B16-F10 and HCT-116 cells	IC_50_ 0.7–3.6 μg/mL	[33,34,35]
Abyssomicin Z2 (**46**)	Other polyketides	*M. rhizosphaerae* TBRC6028	Rhizosphere soil of *Curcuma longa*, Thailand	NCI-H187 and Vero cells	IC_50_ 4.6 and 38.9 μM	[53]
3,4-Dihydrolactucin (**73**)	Sesquiterpene lactone	*Microbispora* sp. AL22	Rhizosphere soil associated with *Alpinia galanga*	MDA-MB-231, HeLa cells	IC_50_ 37.6–75.3 μg/mL	[55,70]
Questiomycin A (**50**)	Alkaloids	*Microbispora* sp. TP-A0184	Soil, Toyama, Japan	U937 cells	IC_50_ 0.2 μg/mL	[57]
Glucosylquestiomycin (**53**)	Alkaloids	*Microbispora* sp. TP-A0184	Soil, Toyama, Japan	U937 cells	IC_50_ 11.2 μg/mL	[57]

IC_50_, half-maximal inhibitory concentration. Only representative cytotoxic activities with quantitative IC_50_ values are summarized.

**Table 2:** Representative antimicrobial activities of selected *Microbispora*-derived metabolites.

Compound	Class	Producing Strain	Source	Key Active Spectrum	Representative Activity	Refs.
Hibarimicins A–D (**2**–**5**)	Quinones	*M. rosea* subsp. *hibaria* TP-A0121	Soil, Toyama, Japan	Gram-positive bacteria	MIC 0.8–12.5 μg/mL	[33,34,35]
Bispolides A1 and B1 (**36**, **39**)	Macrolides	*Microbispora* sp. A34030	Soil, Selangor, Malaysia	MRSA clinical isolates	MIC 1–4 μg/mL	[48]
Glucosylquestiomycin (**53**)	Alkaloids	*Microbispora* sp. TP-A0184	Soil, Toyama, Japan	Bacteria and yeasts	MIC 6.25–50 μg/mL	[57]
Questiomycin A (**50**)	Alkaloids	*Microbispora* sp. TP-A0184	Soil, Toyama, Japan	Bacteria and yeasts	MIC 1.56–25 μg/mL	[57]
Microbisporicin A1/A2 (**70**/**71**)	Peptides	*Microbispora* sp. ATCC PTA-5024	Soil, the Vicuron laboratory collection	Gram-positive pathogens, including MRSA, VISA, and VRE	MIC ≤ 0.13–4 μg/mL	[67]
3,4-Dihydrolactucin (**73**)	Sesquiterpene lactone	*Microbispora* sp. AL22	Rhizosphere soil associated with *Alpinia galanga*	Pathogenic bacteria	MIC 16–64 μg/mL	[55]
Sch 31828 methyl ester (**75**)	Polyacetylene-containing metabolite	*Microbispora* sp. SCC 1438	Soil-derived *actinomycete*	*Candida* spp. and dermatophytes	Geometric mean MICs values of 0.034 and 1 μg/mL, respectively	[71]

MIC, minimum inhibitory concentration; MRSA, methicillin-resistant *Staphylococcus aureus*; VISA, vancomycin-intermediate *Staphylococcus aureus*; VRE, vancomycin-resistant enterococci. Only representative antimicrobial activities with quantitative MIC values are summarized.

**Table 3:** Representative neuroprotective, antiviral, enzyme inhibitory, and other biological activities of selected *Microbispora*-derived metabolites.

Compound	Class	Producing Strain	Source	Activity Type	Representative Activity	Refs.
Chromone analogs **26**, **27**, **29**–**33**	Chromones/chromanones	*Microbispora* sp. TBRC6027	Soil, herbal garden, Thailand	Neuroprotective activity	Cell viability 43.5–53.0% at 1 ng/mL	[17]
Okichromanone (**35**)	Chromones/chromanones	*Microbispora* sp. OK76	Marine sponge, Okinawa, Japan	Antiviral activity	IC_50_ 40 and 59 μM against HSV-1 and HSV-2	[22]
Linfuranone A (**43**)	Other polyketides	*Microbispora* sp. GMKU 363	Thai medicinal plant	Adipogenic activity	Approximately 47% adipocyte differentiation at 50 μM	[50]
1-Hydroxyphenazine (**56**)	Alkaloids	*Microbispora* sp. OK76	Marine sponge, Okinawa, Japan	Antiviral activity	IC_50_ 86 and 123 μM against HSV-1 and HSV-2	[22]
Anthranilic acid (**61**)	Alkaloids	*M. hainanensis* CSR-4	Rhizosphere soil of *Zingiber montanum*	Spore germination inhibition	IC_50_ 50 μg/mL against *Streptomyces* spore germination	[60]
Cochinmicins I–III (**63**–**65**)	Peptides	*Microbispora* sp. ATCC 55140	Soil, Cochin, India	ET-1 receptor binding	IC_50_ 0.2–22 μM; K_i_ 0.12–3.1 μM	[61,62,63]
Propeptin (**68**)	Peptides	*Microbispora* sp. SNA-115	Soil, Kanagawa, Japan	PEP inhibition	IC_50_ 1.1 μM; K_i_ 0.7 μM	[64,65]
Propeptin-2 (**69**)	Peptides	*Microbispora* sp. SNA-115	Soil, Kanagawa, Japan	PEP inhibition	K_i_ 1.5 μM	[66]
2α-hydroxy-8(14),15-pimaradien-17,18-dioic acid (**76**)	Other types	*M. hainanensis* CSR-4	Rhizosphere soil of *Zingiber montanum*	Neuroprotective and anti-rhAChE activities	Cell viability 88.6% under serum deprivation; IC_50_ 96.9 ± 2.3 μg/mL against rhAChE	[58]
microbispofurans A–C (**79**–**81**)	Other types	*Microbispora* sp. RD004716	Plant root	Plant growth-promoting	Seedling growth 62–102% of control at 10 μM	[24]

IC_50_, half-maximal inhibitory concentration; K_i_, inhibition constant; ET-1, endothelin-1; PEP, prolyl endopeptidase; rhAChE, recombinant human acetylcholinesterase; HSV, herpes simplex virus. Only representative biological activities other than antimicrobial and cytotoxic effects are summarized.

## 3. Genomic Insights into the Biosynthetic Potential of *Microbispora*

### 3.1. Conserved Genomic Features of the Microbispora Genome

Comparative analysis of the genome sequences summarized in Table S1 indicates that *Microbispora* species share relatively conserved genomic features. Among the 83 publicly available genome assemblies, genome sizes generally range from approximately 7.0 to 10.0 Mbp, with most strains exhibiting genome sizes of 8–9 Mbp. The genomic G+C content is also relatively conserved, ranging from approximately 65% to 73%, with most strains showing values around 70–72%. The relatively large genome sizes and high G+C contents represent characteristic genomic features of the genus and are consistent with the typical genomic composition of moderately high-G+C filamentous actinobacteria [72,73,74]. These genomic characteristics may provide substantial genetic capacity for diverse metabolic functions, particularly specialized metabolite biosynthesis.

Representative genome sequences further illustrate the conserved genomic features of this genus. The plant-associated strain *Microbispora* sp. GKU 823, isolated from sugarcane roots in Thailand, possesses an estimated 9.4 Mbp draft genome with a G+C content of 71.3% and 9248 predicted coding sequences, while genome mining revealed diverse secondary metabolite BGCs, including those associated with polyketides, nonribosomal peptides, terpenes, bacteriocins, and siderophores, highlighting the considerable biosynthetic potential of this strain [75]. These values differ slightly from those listed in Table S1, possibly because different genome assembly and annotation records were used; moreover, the reported coding-sequence count and total annotated-gene count are not directly equivalent.

The linfuranone A-producing strain *Microbispora* sp. GMKU 363 contains a 7,820,463 bp genome with a G+C content of 69.6%. This genome harbors multiple PKS- and NRPS-related biosynthetic regions, including the gene cluster responsible for linfuranone A production [76]. In addition, *Microbispora* sp. ATCC PTA-5024, a well-known producer of the lantibiotic NAI-107, possesses a genome of 8.5 Mbp with a G+C content of 71.2% and 7860 protein-coding genes. antiSMASH analysis identified approximately 20 secondary metabolite biosynthetic gene clusters in this strain, in addition to the *mlb* gene cluster responsible for NAI-107 biosynthesis [77]. Consistent with these observations, genomic characterization of *Microbispora* sp. KK1-11 further revealed multiple secondary metabolite biosynthetic gene clusters, providing additional genomic evidence for the biosynthetic potential of this genus [78]. Together, these studies indicate that the conserved genomic architecture of *Microbispora* is associated with substantial biosynthetic capacity for specialized metabolite.

### 3.2. Biosynthetic Gene Cluster Distribution Reflects Chemical Diversity

**Figure 8:**
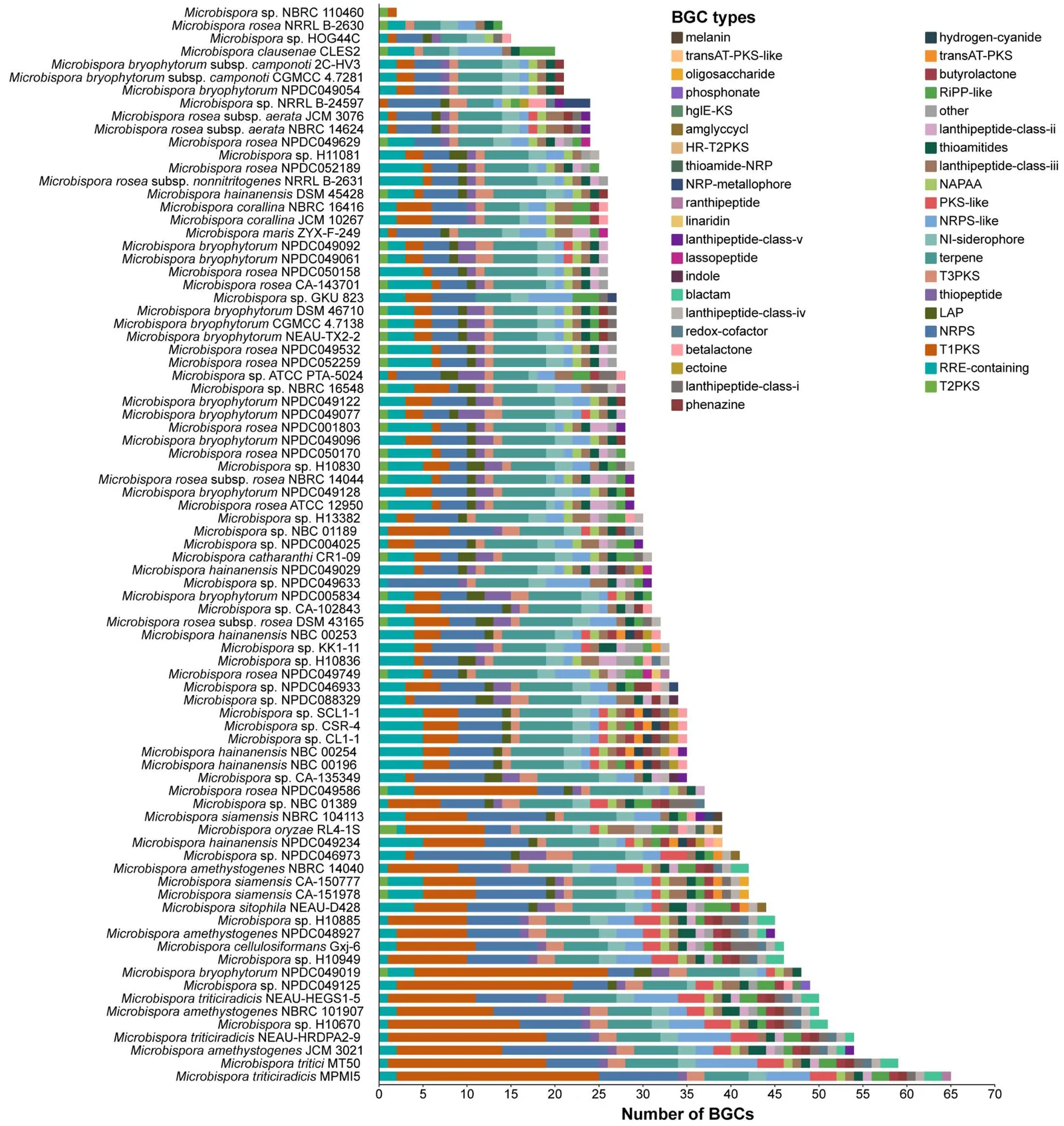
Distribution of different BGC classes across 83 publicly available *Microbispora* genome assemblies. BGCs were predicted from genome assemblies retrieved from the NCBI Assembly database using antiSMASH v8.0 with default parameters. The dataset included GenBank and RefSeq records, and assembly accession numbers are listed in Table S1. Abbreviations: NRPS, nonribosomal peptide synthetase; T1PKS, type I polyketide synthase; T2PKS, type II polyketide synthase; *trans*-AT PKS, *trans*-acyltransferase polyketide synthase; RiPP, ribosomally synthesized and post-translationally modified peptide.

Although individual genome studies have demonstrated the biosynthetic potential of several *Microbispora* strains, the overall distribution and diversity of BGCs across the genus remain poorly characterized. Here, comparative genome mining of 83 publicly available *Microbispora* genomes revealed extensive and heterogeneous biosynthetic potential across the genus. The number of predicted BGCs varied considerably among strains, ranging from several clusters to more than 60 clusters per genome (Figure 8; Table S1). These results demonstrate that *Microbispora* genomes contain rich and diverse biosynthetic repertoires, providing a genomic basis for the structural diversity of metabolites reported from this genus.

The major BGC classes identified in *Microbispora* genomes include nonribosomal peptide synthetase (NRPS), polyketide synthase (PKS, including type I, type II, type III, and *trans*-AT PKS), ribosomally synthesized and post-translationally modified peptide (RiPP), terpenes, siderophores, and other specialized metabolite pathways [79]. Several BGC types are widely distributed among sequenced strains, whereas others show clear strain-specific or lineage-associated patterns. For example, NRPS and PKS clusters are commonly detected across the genus, consistent with the abundance of peptide and polyketide metabolites isolated from *Microbispora*. RiPP-associated clusters are also detected in several genomes, including the lanthipeptide biosynthetic machinery responsible for microbisporicin production, a potent lantibiotic active against multidrug-resistant Gram-positive pathogens [67].

The distribution of specific BGC classes shows a clear correspondence with the chemical diversity observed among isolated metabolites. NRPS clusters are widespread but vary considerably in abundance among strains, suggesting different capacities for producing complex peptide-based natural products, such as cochinmicins and propeptin-related compounds [61,62,63,64,65,66]. Similarly, PKS-associated clusters exhibit substantial diversity, with type I PKS and *trans*-AT PKS pathways providing the genetic basis for the biosynthesis of structurally complex polyketides [80,81], including bispolides and linfuranone-related metabolites [48,50]. Type II PKS clusters are frequently observed in *Microbispora rosea*-related strains, consistent with the predominance of quinone-type metabolites, such as hibarimicins, reported from this lineage [33,34,35].

Beyond PKS and NRPS pathways, other specialized BGCs further expand the chemical landscape of *Microbispora*. Siderophore-associated clusters are broadly distributed and may contribute to microbial competitiveness under iron-limited conditions [82]. Terpene and other specialized metabolite clusters, although less directly linked to characterized compounds, represent additional reservoirs of unexplored chemical diversity. Notably, several predicted BGCs have not yet been linked to characterized metabolites, suggesting the presence of cryptic biosynthetic systems capable of generating previously undiscovered metabolites.

Collectively, genome mining demonstrates that *Microbispora* possesses a highly diverse and strain-specific biosynthetic landscape. The correspondence between BGC distribution and known metabolite classes highlights the value of integrating genomic prediction with chemical analysis, providing an effective strategy for accelerating the discovery of novel bioactive natural products from this underexplored actinobacterial genus.

### 3.3. Genome Mining as a Roadmap for Future Discovery

The expanding genomic resources of *Microbispora* highlight this genus as a promising reservoir of unexplored biosynthetic diversity. The integration of strain-specific BGC profiles, phylogenetic relationships, and associations between predicted biosynthetic pathways and known metabolite classes provides a rational strategy for targeted natural product discovery. Several characterized metabolites, including hibarimicins, microbisporicin, and linfuranone-related compounds, demonstrate the feasibility of genome-guided approaches for linking BGCs to chemically diverse metabolites.

Future genome mining efforts should prioritize strains with abundant or rare BGCs, particularly those containing BGC classes such as *trans*-AT PKS, type II PKS, RiPP, or thioamide NRP clusters. However, the presence of a predicted BGC does not necessarily indicate active metabolite production, as many pathways remain silent under standard cultivation conditions [83]. Therefore, genome-guided prioritization should be combined with strategies for activating cryptic pathways, including one strain–many compounds (OSMAC) cultivation, co-culture, chemical elicitation, and heterologous expression of selected BGCs [84]. Integration of these approaches with metabolomics-based dereplication and molecular networking will facilitate the discovery of previously inaccessible bioactive metabolites from *Microbispora* [85].

## 4. Conclusion and Future Perspectives

**Figure 9:**
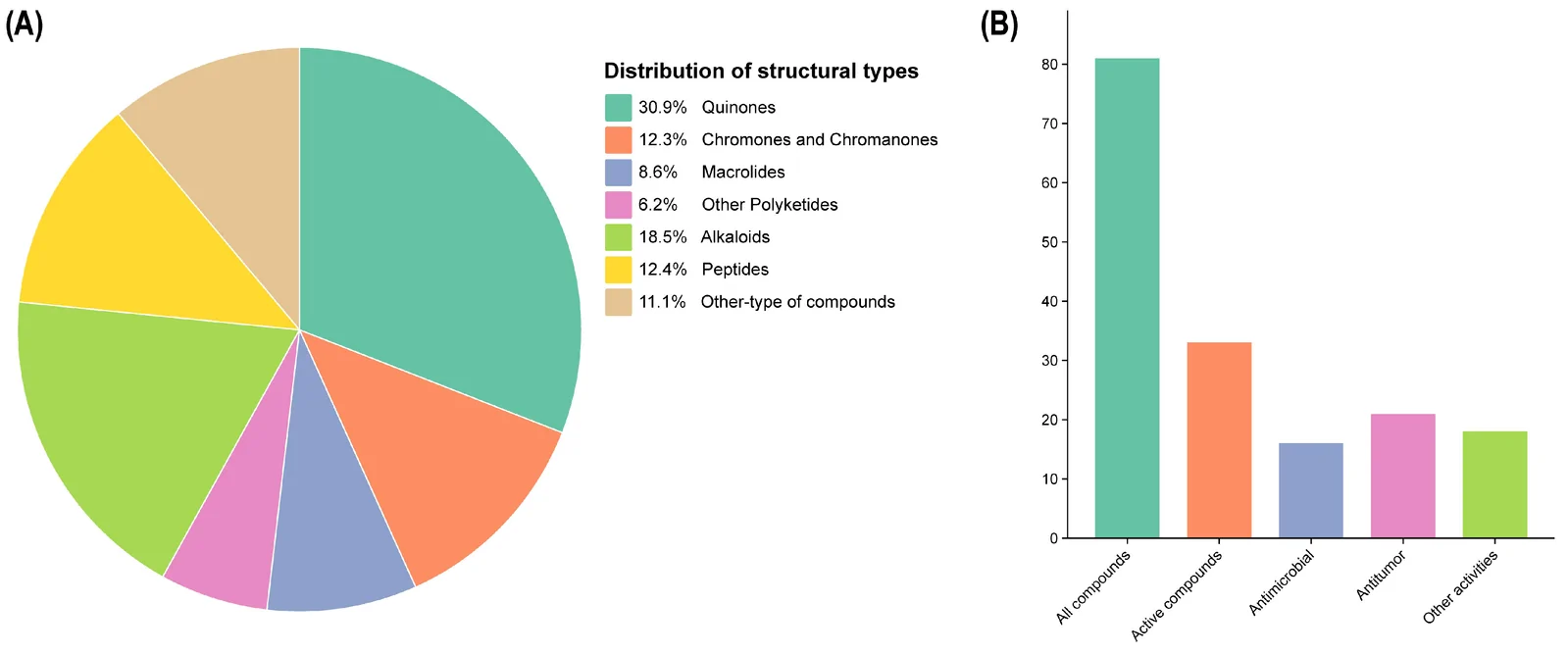
Overview of reported natural products from the genus *Microbispora*. (**A**) Distribution of structural classes among 81 reported metabolites. (**B**) Distribution of biological activity categories based on reported assays. Note that one compound may be assigned to multiple biological activity categories.

To date, eighty-one secondary metabolites have been reported from the genus *Microbispora*. These compounds were predominantly discovered through conventional fermentation-based screening, with quinones representing one of the most abundant structural class (Figure 9A), reflecting both the biosynthetic capacity of the genus and historical research preferences toward aromatic metabolites. As summarized in Figure 9B, *Microbispora*-derived metabolites exhibit diverse biological activities, including antimicrobial, cytotoxic, neuroprotective, antiviral, and enzyme inhibitory effects. However, despite extensive structural studies, the absolute configurations of several metabolites remain incompletely established, highlighting opportunities for further stereochemical investigation.

*Microbispora* has proven to be a valuable source of structurally diverse and biologically active natural products. Representative metabolites, including microbisporicin (**70**, **71**) and the Src kinase-inhibiting quinones (**1**–**5**), represent promising bioactive scaffolds owing to their notable biological activities and distinctive mechanisms of action. Nevertheless, most *Microbispora*-derived compounds remain at the early discovery stage, and their pharmacokinetic properties, in vivo efficacy, and safety profiles are largely unexplored. Furthermore, some reported biological activities, particularly those with micromolar-range potency, require further optimization before potential therapeutic applications can be considered.

Genome mining analyses have revealed extensive cryptic biosynthetic potential within *Microbispora*, indicating that the currently characterized metabolites represent only a small fraction of the chemical diversity encoded by this genus. Future studies should integrate genome mining, metabolomics-guided dereplication, synthetic biology, and activation of silent biosynthetic pathways to access previously unexplored metabolites. In addition, systematic investigation of structure–activity relationships and evaluation in relevant biological models will be essential for translating *Microbispora*-derived natural products into valuable bioactive leads.

Although most reported *Microbispora*-derived metabolites have been obtained from terrestrial environments, marine-associated and other underexplored habitats represent promising sources of additional chemical diversity. Marine-associated isolates have shown promising biosynthetic potential, as exemplified by sponge-associated *Microbispora* sp. OK76, which yielded structurally unique metabolites, including okichromanone and 1-hydroxyphenazine. In addition, the Antarctic-associated strain *Microbispora aerata* IMBAS-11A, isolated from penguin excrements, produced a sulfur-containing indole alkaloid and a diketopiperazine derivative. Newly described marine-associated species, including *Microbispora hainanensis* and marine animal-associated *Microbispora maris*, further highlight the unexplored diversity of marine-derived *Microbispora*. Future exploration of marine, coastal, and other underexplored habitats, combined with genome-guided and metabolomics approaches, will provide opportunities to uncover additional biosynthetic pathways and expand the chemical space of *Microbispora*.

Overall, *Microbispora* represents an underexplored reservoir of actinobacterial chemical diversity and biosynthetic potential. Integrating advanced cultivation strategies, genome-guided discovery, metabolomics, and functional biosynthetic engineering will be essential for accessing its hidden chemical repertoire. Future efforts should also focus on activating silent biosynthetic gene clusters, improving heterologous expression strategies, and establishing stronger links between genomic predictions and experimentally validated metabolites. Such multidisciplinary approaches will not only facilitate the discovery of structurally unique bioactive metabolites from *Microbispora* but also provide valuable insights into the untapped potential of rare actinomycetes in the post-genomic era.

## Data Availability

No new data were created or analyzed in this study. Data sharing is not applicable to this article.

## Supplementary Materials

The following supporting information can be downloaded at https://www.mdpi.com/article/10.3390/md24080263/s1, Table S1: Genomic characteristics and biosynthetic potential of 83 publicly available *Microbispora* genome assemblies included in this study.
